# How Nutrient Deficiencies Impact the Oral Mucosa and How to Manage Them: A Narrative Review

**DOI:** 10.3390/nu18172818

**Published:** 2026-08-28

**Authors:** Patrycja Starzyńska, Anna Sokołowska, Kinga Bociong, Sebastian Kłosek

**Affiliations:** 1 Department of Oral Mucosal and Periodontal Diseases, Medical University of Lodz, 92-213 Lodz, Poland; 2 Department of General Dentistry, Medical University of Lodz, 92-213 Lodz, Poland; 3 Department of Oral Pathology, Medical University of Lodz, 92-213 Lodz, Poland; sebastian.klosek@umed.lodz.pl

**Keywords:** malnutrition, nutrient deficiencies, oral mucosa, recurrent aphthous stomatitis, atrophic glossitis, burning mouth syndrome, lingual lesions, oral lichen planus, angular cheilitis, oral candidiasis

## Abstract

Background: Malnutrition and micronutrient deficiencies may impact the oral mucosa even before any other symptoms become clinically obvious. This makes the oral cavity an important site for early detection of nutritional imbalance. Across the literature review, the most documented vitamin is vitamin B12. Other widely reported nutrients are iron, folate, and zinc. Less consistently documented are vitamins A, C, and D. Their correlation with recurrent aphthous stomatitis (RAS), atrophic glossitis, angular cheilitis, burning mouth symptoms (BMS), mucosal erythema, and oral candidiasis (OC) is brought to light. This review collects evidence on how nutritional deficiencies influence epithelial turnover, immune competence, oxidative balance, and mucosal repair to understand the pathogenesis of oral mucosal diseases. Methods: This narrative review draws on literature searches of major databases using terms related to malnutrition, nutritional deficiencies, and oral mucosal diseases. Results: The evidence is strongest for RAS and atrophic glossitis, where several observational studies and recent systematic reviews associate hematinic deficiencies with disease presence and, in some cases, symptom improvement after performing replacement therapy. Zinc deficiency has also been linked to recurrent ulceration, burning mouth syndrome, and epithelial alterations, while vitamin B12 deficiency may present with glossitis, BMS, angular cheilitis, mucosal ulceration, and lingual linear lesions even with the absence of systemic anemia. Oral candidiasis is more common in malnourished patients due to reduced host resistance and diminished salivary protection, especially in older or medically compromised patients. Conclusions: Although causality cannot be assumed from all included studies, the literature suggests that unexplained oral mucosal lesions, especially when they are multiple or persistent, warrant nutritional screening as part of a comprehensive oral medicine assessment.

## 1. Introduction

Malnutrition has been recognized as an important factor in the development and progression of oral mucosal diseases for a long time now [1,2,3,4]. The oral mucosa is a rapidly renewing tissue that depends on adequate macro- and micronutrient availability to maintain epithelial integrity, vascular support, neurosensory function, and host defense [5,6,7]. For this reason, nutritional deficiencies could become clinically visible in the mouth earlier than in other tissues, especially when deficits impair DNA synthesis, epithelial maturation, collagen formation, oxidative balance, or immune regulation [5,6,7,8].

In clinical practice, malnutrition-related oral changes rarely appear as a single lesion. Instead, they have the tendency to create clusters of symptoms that include oral soreness, mucosal erythema, recurrent ulceration, depapillation of the tongue, angular fissuring, candidal colonization, dysgeusia, and burning sensations [9,10,11]. These manifestations overlap with inflammatory, autoimmune, infectious, gastrointestinal, and hematologic disorders [9,10,11,12,13]. For this reason, nutritional causes are often underrecognized unless laboratory screening is deliberately incorporated into the diagnostic process [9,10,11,14,15]. The main goal of this review is to summarize the literature from the last 10 years and to underline for practitioners the importance of seeing the patient systemically, rather than focusing solely on oral mucosa changes.

The published research is particularly rich in studies of recurrent aphthous stomatitis, atrophic glossitis, burning mouth syndrome, oral lichen planus, oral candidiasis, and related oral mucosal diseases with documented hematinic abnormalities. It also includes broader nutritional and pathophysiologic literature relevant to the epithelial barrier regulation, oxidative stress, and oral manifestations of systemic disease [6,7,16,17,18]. Based on these sources, this review summarizes the main clinical manifestations of oral mucosal malnutrition, the most frequently implicated deficiencies, the biologic mechanisms that may connect nutritional imbalance and mucosal disease, and the practical consequences for oral diagnosis and patient management.

## 2. Methodology

### 2.1. Searching Strategy

This article is a narrative review based on the literature search across the databases PubMed/MEDLINE, Google Scholar, Scopus, and National Library of Medicine using combinations of keywords: “malnutrition”, “nutritional deficiencies”, “oral mucosal manifestations”, “oral mucosa conditions”, “oral mucosa diseases”, “oral mucosa outcome”. Articles published between 2015 and 2026 were screened for relevance with additional key papers from 2010 to 2014 incorporated for conceptual context; older articles from 1973 to 1985 were included to provide historical background. The strongest evidence concerns recurrent aphthous stomatitis, atrophic glossitis, burning mouth syndrome, oral lichen planus, and oral candidiasis, associated with iron, folate, vitamin B12, zinc, and other micronutrients.

The reference lists of relevant reviews and primary studies were also manually screened to identify additional eligible publications that were not captured by the electronic search. The narrative review was based mainly on English-language, peer-reviewed journal articles, supplemented where appropriate by additional relevant sources. This approach was used to provide broad coverage of both clinical and mechanistic evidence concerning the diet–oral mucosa health relationship, recognizing that summaries are lacking. This article aims to summarize clinical content from the latest sources on how to diagnose and manage oral mucosa conditions caused by nutrient deficiencies.

The main goals of this narrative review are to summarize existing knowledge on a topic, provide a clinical context, interpret and integrate findings from diverse studies, and offer a coherent overview that guides future research and practitioners. Accordingly, greater weight is given to systematic reviews, meta-analyses, larger case–control or cohort studies, and classic reports that have shaped the present understanding of deficiency-related oral disease, while isolated case reports are primarily used to illustrate clinically distinctive phenotypes.

The review focuses on evidence-based clinical trials, observational research, systematic reviews, meta-analyses, and selected mechanistic studies addressing inflammatory, oxidative, and microbial processes. At the same time, experimental in vitro and animal data were considered only when they helped clarify mechanisms relevant to human oral physiology.

### 2.2. Inclusion and Exclusion Criteria

A targeted narrative review approach was used to identify publications relevant to nutritional deficiencies and oral mucosal diseases. The bibliography included human studies, systematic reviews, narrative reviews, case–control studies, cohort studies, cross-sectional studies, case series, and clinically informative case reports addressing oral mucosal conditions associated with malnutrition or micronutrient deficiency. Eligible sources mainly focused on the most common oral mucosal manifestations. Studies were included when they examined micronutrient deficiencies and immune mechanisms relevant for oral mucosal pathology. Priority was given to peer-reviewed articles with clear clinical relevance or diagnostic and therapeutic implications for oral medicine practice.

Studies were excluded if they did not address oral mucosal outcomes, did not evaluate nutritional status or deficiency-related mechanisms, or focused exclusively on non-mucosal conditions such as dental caries or general systemic nutrition without oral mucosal relevance. Animal-only studies were excluded from the main clinical synthesis, although selected experimental studies were considered when they provided important mechanistic support for the Section 3.1. Editorials, conference abstracts without sufficient data, and duplicate publications were excluded from the core evidence base. Articles focused primarily on oral potentially malignant disorders or oral cancer were not used as primary evidence for the diagnosis of deficiency-related mucosal lesions.

## 3. Pathophysiology and Selected Conditions

### 3.1. Pathophysiology

The oral mucosa is a biologically dynamic tissue that depends on adequate nutrient supply for epithelial maturation, wound repair, neurosensory function, and host defense [6,7,8,19,20]. The oral epithelium turns over rapidly and therefore depends on adequate supplies of iron, folate, and vitamin B12 for DNA synthesis and normal maturation. Deficiency of these hematinics can lead to epithelial atrophy, papillary loss on the tongue, reduced healing ability, and increased susceptibility to ulceration or soreness [5,19,21,22]. This biologic model is consistent with the recurring observation that glossitis, stomatitis, and recurrent aphthous lesions coexist with anemia, microcytosis, macrocytosis, hyperhomocysteinemia, or isolated hematinic depletion in oral medicine cohorts [11,21,23,24].

Zinc contributes to epithelial growth, wound healing, keratinization, and immune regulation [8,20]. Experimental work in zinc-deficient animals showed early parakeratosis, epithelial thickening, vascular changes, and increased mitotic activity in buccal mucosa. In human studies, zinc deficiency has been identified as a potential contributor to recurrent aphthous stomatitis, burning mouth syndrome, geographic tongue, and erosive oral lichen planus. It confirms the concept that altered zinc homeostasis can modify both mucosal structure and inflammatory responses [25,26,27,28].

Vitamins with antioxidant or immunomodulatory functions may additionally influence oral mucosal resilience. The literature includes studies linking recurrent aphthous disease to oxidative stress and altered antioxidant markers. Broader reviews suggest that vitamins A, C, D, and E can affect epithelial repair, immune function, and mucosal barrier homeostasis [8,20,29,30,31,32]. Salivary gland function is another relevant pathway, because protein-energy malnutrition in childhood has been associated with reduced salivary flow later in life, which may compromise the immune system and increase mucosal vulnerability [7,12,33].

**Figure 1:**
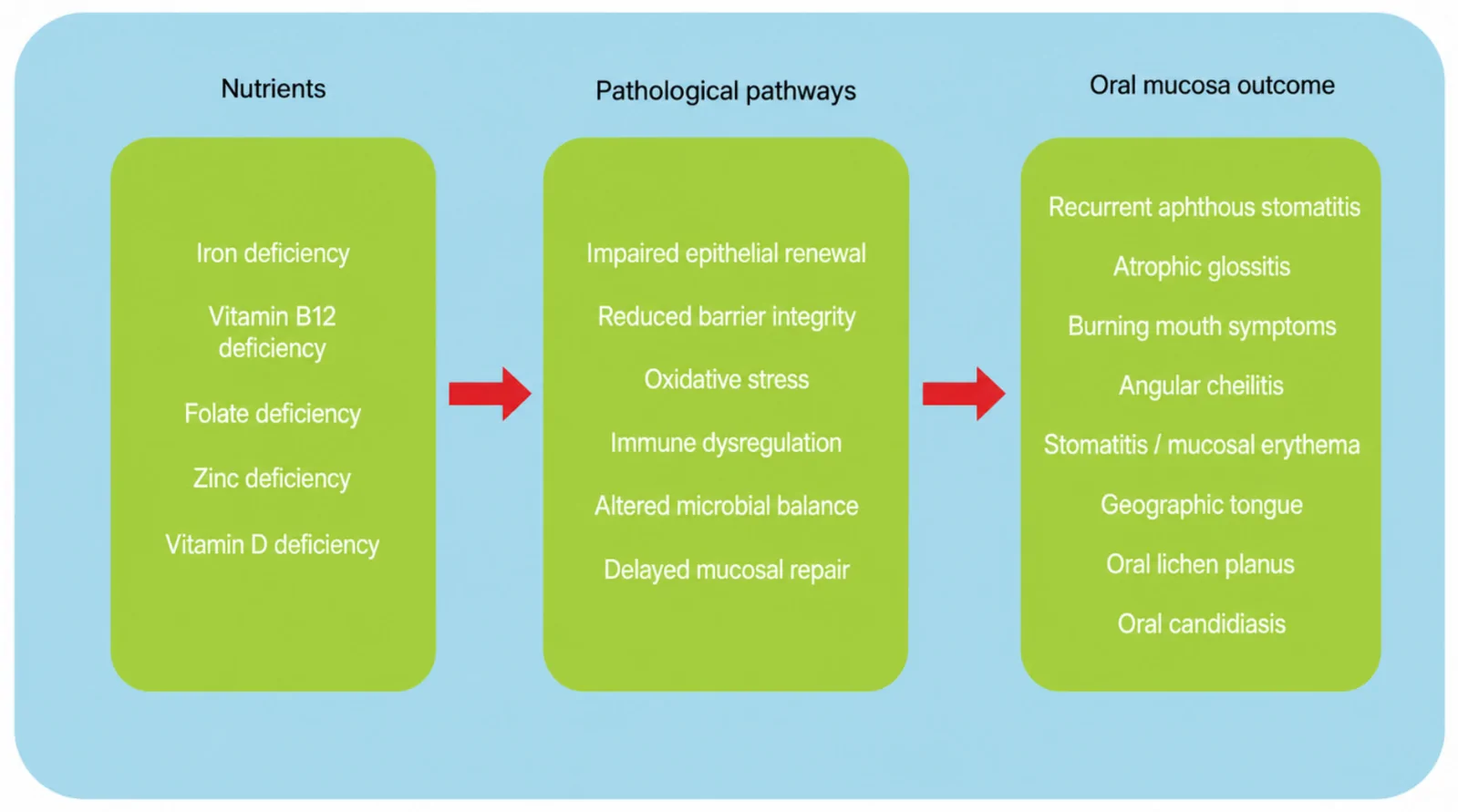
The schematic representation of the pathophysiological processes of selected conditions.

Figure 1 is a simplified schematic representation linking selected nutritional deficiencies with the principal pathological pathways and oral mucosal outcomes discussed in this review. The first column identifies iron, vitamin B12, folate, zinc, and vitamin D deficiencies as the main nutritional factors considered. These deficiencies may contribute to impaired epithelial renewal, reduced mucosal barrier integrity, oxidative stress, immune dysregulation, altered microbial balance, and delayed mucosal repair, as shown in the central column [6]. The final column presents the principal oral mucosal conditions associated with these pathways, including recurrent aphthous stomatitis, atrophic glossitis, burning mouth symptoms, angular cheilitis, stomatitis or mucosal erythema, geographic tongue, oral lichen planus, and oral candidiasis. The arrows indicate a conceptual relationship between nutritional imbalance, biological disturbances in the oral mucosa, and the clinical manifestations described in the subsequent sections. These relationships are not intended to imply that each deficiency causes every listed condition, because the clinical expression of oral disease is influenced by multiple interacting factors, including immune, microbial, local, and systemic conditions [34].

### 3.2. Recurrent Aphthous Stomatitis (RAS)

Recurrent aphthous stomatitis is the most extensively represented clinical entity in the sources and the clearest example of a mucosal disorder with a substantial nutritional dimension. Although its etiology is multifactorial and includes immune, microbial, genetic, and psychosocial components, multiple studies in the reference set report associations between recurrent aphthous stomatitis and deficiencies of iron, vitamin B12, folate, and zinc, and hemoglobin-related indices [5,35,36].

Older but still influential reports found that a meaningful subset of patients with recurrent aphthae had vitamin B12, folate, or iron deficiency, and that targeted replacement therapy was followed by complete remission or marked improvement more often than symptomatic treatment alone [21]. More recent observational studies continue to support this relationship. Although Ślebioda et al. reported no differences in serum zinc status between patients with recurrent aphthous stomatitis and the control group, other studies have identified low zinc levels as an important risk factor [25,27,28,37]. At the same time, other studies found differences in antioxidant vitamins, oxidative stress markers, and hematological parameters between affected patients and controls [17,38,39,40]. There are also systematic reviews and meta-analyses that indicate that patients with recurrent aphthous stomatitis are more likely than controls to have lower vitamin B12, ferritin, folate, iron, and hemoglobin values, strengthening the clinical relevance of hematinic screening [24,41,42].

A practical challenge is that deficiency-associated aphthous disease is not clinically unique in appearance. The ulcers resemble ordinary minor or major aphthae, and the nutritional contribution may only become apparent when recurrent ulceration is accompanied by pallor, glossal discomfort, diffuse erythema, fatigue, gastrointestinal history, autoimmune gastritis, malabsorption, or poor dietary intake [9,36,43]. Accordingly, there is still a need for laboratory investigation in recurrent, severe, treatment-resistant, or clinically atypical cases.

Further research shows that the nutritional contribution to recurrent aphthous stomatitis may extend beyond the classical hematinics. Vitamin D was reported to be lower in some case–control studies, and oxidative stress-focused papers indicate a biological environment in which deficient antioxidant protection may facilitate mucosal breakdown [44,45,46,47]. Nevertheless, the most consistent and clinically actionable evidence continues to focus on iron, folate, vitamin B12, zinc, and anemia-related parameters [10,24,40].

It should also be noted that, beyond nutritional factors, certain local irritants have been implicated in recurrent aphthous stomatitis. In particular, sodium lauryl sulfate (SLS), a detergent commonly used in oral hygiene products, has been investigated as a potential aggravating factor in susceptible individuals. A systematic review of randomized controlled trials concluded that SLS-free dentifrices, compared with SLS-containing products, significantly reduced the number of ulcers, duration of ulceration, number of episodes, and ulcer pain in patients with recurrent aphthous stomatitis [48]. Subsequent scoping reviews and umbrella reviews have confirmed these findings, although they emphasize that the available evidence remains limited and based on a small number of crossover trials [49,50]. Some authors have suggested that SLS may exert a denaturing effect on the oral mucin layer, thereby exposing the underlying epithelium and potentially facilitating ulcer formation or delaying healing [50,51]. While these observations indicate that SLS avoidance may be a useful adjunctive measure in selected patients, this topic lies outside the primary scope of the present review, which focuses on systemic malnutrition and micronutrient deficiencies as contributors to oral mucosal disease.

### 3.3. Atrophic Glossitis

Atrophic glossitis is one of the most characteristic manifestations of oral mucosal malnutrition and is repeatedly linked to hematinic deficiency [22,52,53]. Clinically, it presents as depapillation of the dorsal tongue, often visible as a smooth, glossy, erythematous, and tender surface. Patients may describe it as burning, sore, or unusually sensitive to spicy food.

The strongest evidence comes from the work of Chiang et al., who examined large clinical cohorts and consistently documented how important it is to run a full blood count to establish if the patient has anemia, iron deficiency, vitamin B12 deficiency, folate deficiency, or hyperhomocysteinemia. In patients with atrophic glossitis, gastric parietal cell antibodies should be examined as well [54,55,56]. These studies are important because they move the discussion beyond isolated case descriptions and show that atrophic glossitis is often a marker of an underlying systemic disorder, particularly pernicious anemia, iron depletion, or autoimmune gastric dysfunction. Atrophic glossitis may appear alone or in combination with angular cheilitis, mucosal erythema, recurrent ulceration, or burning mouth symptoms [21,39,57,58]. The literature additionally emphasizes that oral symptoms can precede overt hematologic abnormalities, meaning that a normal or only mildly altered blood count does not fully exclude nutritionally mediated glossal disease. This lesion is clinically important because it has a relatively strong pathophysiologic fit with deficiency biology. Iron, folate, and vitamin B12 are necessary for epithelial renewal, and deficiency leads to papillary atrophy, mucosal thinning, and sensory discomfort [59,60]. In addition, atrophic glossitis may reflect malabsorption syndromes, pernicious anemia, or chronic gastrointestinal disease, so the tongue can serve as a visible marker of wider nutritional compromise [22].

From a clinical perspective, atrophic glossitis should be considered within a broader differential diagnosis of lingual and oral burning conditions. Similar lingual changes and symptoms may occur in patients with hyposalivation, oral candidiasis, and oral lichen planus, as well as in those with local irritants, contact stomatitis, or neuropathic pain syndromes. Hyposalivation, whether related to medications, Sjögren’s syndrome, or systemic disease, can lead to a smooth, erythematous, and sore tongue that closely mimics deficiency-related glossitis. Oral candidiasis, particularly the erythematous or atrophic form, may present with depapillation, burning, and diffuse erythema, often in the context of denture wearing, xerostomia, or immunosuppression. Oral lichen planus, an immune-mediated mucosal disease, can involve the tongue with erythematous and erosive lesions that may be misinterpreted as nutritional glossitis, especially when coexisting hematinic abnormalities are present [58,61,62,63]. Therefore, a careful clinical examination, review of medications, assessment of salivary function, and, when indicated, mycological and histopathological evaluation are essential to distinguish true nutritional glossitis from these alternative or coexisting conditions [10].

### 3.4. Burning Mouth Symptoms (BMS)/Glossodynia

BMS are a diagnostic challenge. They may reflect primary burning mouth syndrome, secondary neuropathic change, xerostomia, candidiasis, endocrine disorders, or nutritional deficiency [10]. The literature nevertheless suggests that hematinic depletion is common enough in patients with burning mouth complaints to justify systematic evaluation, especially when symptoms coexist with mucosal erythema, depapillation, angular fissuring, or dysgeusia [63].

A large series from the Journal of the Formosan Medical Association reported anemia, iron deficiency, vitamin B12 deficiency, folate deficiency, hyperhomocysteinemia, and gastric parietal cell antibody positivity in substantial subsets of patients with burning mouth syndrome. Other papers also describe zinc deficiency as a potential cause of burning mouth symptoms, with zinc replacement showing therapeutic benefit in some patients [64]. Supplement-based studies involving vitamin B complex and zinc further support the idea that a clinically meaningful subset of burning mouth presentations may be nutritionally influenced [58,65]. Importantly, some patients initially labeled as having idiopathic burning mouth disorder were later found to have atrophic glossitis or vitamin B12 deficiency-related mucosal disease [64]. This reinforces the need to distinguish primary pain syndromes from secondary deficiency-associated conditions, because the latter may improve substantially when the underlying nutritional abnormality is corrected.

### 3.5. Angular Cheilitis and Mucosal Erythema

Angular cheilitis and nonspecific stomatitis are common but often underestimated manifestations of nutritional deficiency. They may present as erythematous fissuring at the corner of the mouth, usually painful, secondary candidal colonization, or diffuse oral soreness without a specific lesion pattern [66].

The reviewed articles include single-center clinical data showing that patients who presented with burning mouth syndrome, angular cheilitis, recurrent aphthous stomatitis, papillary atrophy of the tongue, or mucosal erythema frequently had iron, vitamin B12, or folate deficiency [11]. The case-based literature further illustrates how combined deficiencies can lead to prominent mucocutaneous involvement. For example, a combined zinc and vitamin B6 deficiency after gastric bypass was associated with diffuse erythematous rash, angular cheilitis, and glossitis. These conditions improved with replacement therapy [10]. Such reports remind clinicians that oral commissural lesions may reflect mixed nutritional disorders, malabsorption, or post-surgical states rather than isolated local infections [67,68,69].

Angular cheilitis should also be interpreted within a broader differential diagnosis. Similar lesions at the oral commissures may be caused or exacerbated by local factors such as poorly fitting dentures, excessive lip licking, perioral dermatitis, irritant or allergic contact cheilitis, and secondary infection with Candida or Staphylococcus species [70]. In older adults, angular cheilitis is frequently associated with xerostomia, reduced vertical dimension of occlusion, and denture-related stomatitis, which create a moist, macerated environment conducive to microbial overgrowth [71]. Nutritional deficiencies, particularly of iron and B vitamins, may predispose to or perpetuate angular cheilitis, but they often coexist with these local and mechanical factors rather than acting as the sole cause. Consequently, management should address both potential systemic contributors and local predisposing conditions, including denture adjustment, topical antifungal or antibacterial therapy [72].

### 3.6. Oral Lichen Planus and Related Inflammatory Mucosal Disease

Oral lichen planus is not typically classified as a deficiency disorder, yet the literature contains many investigations into its nutritional correlates [61,62]. Several case–control and cohort studies report hematinic deficiencies, lack of vitamin B12, and zinc level changes in oral lichen planus. The findings are less uniform than in previous conditions [73,74], meaning the evidence should be interpreted carefully. Some studies identified lower serum zinc or vitamin B12 in symptomatic or erosive oral lichen planus, while others found no major differences in vitamin panels between patients and controls [75,76]. More recent work indicates that oral lichen planus cohorts may exhibit increased rates of anemia, hematinic deficiency, hyperhomocysteinemia, and autoimmune serology. It may suggest that nutritional compromise coexists with immunologic disease and possibly modulates symptoms rather than being a sole cause [74,77].

From a clinical perspective, oral lichen planus is primarily an immune-mediated disease, and nutritional deficiencies should be regarded, at most, as possible associated or modifying factors rather than as a central etiological explanation. Therefore, persistent soreness, erosive lesions, or refractory symptoms may warrant hematologic assessment as part of a more comprehensive oral medicine work-up [77,78].

Reports on zinc therapy for symptomatic oral lichen planus also suggest that correcting deficiency or marginal insufficiency may be associated with a favorable outcome in selected patients [75].

### 3.7. Geographic Tongue and Lingual Morphologic Change

Lingual lesions are frequently discussed in the literature on deficiency. The tongue is highly sensitive to disturbances in epithelial maturation. Studies on geographic tongue and linear lingual lesions have examined their relationship with zinc and vitamin B12 status [79,80].

In geographic tongue, available evidence is weaker and less consistent than for atrophic glossitis. One study shows lower levels of salivary zinc in affected patients without other deficiencies of iron or vitamin B12 [79]. This suggests that alterations may exist, but further research is needed. By contrast, linear lingual lesions have been described as a strong clinical sign of severe vitamin B12 deficiency, adding to the wider literature that views the tongue as a sensitive marker of cobalamin depletion [39,81]. These observations are clinically useful because lingual morphology may offer visual clues when deficiency remains otherwise unsuspected. A smooth tongue, linear erythematous lesions, papillary loss, tenderness, or altered surface texture should therefore raise the possibility of hematinic deficiency, especially when associated with symptoms such as burning, dysgeusia, paresthesia, or systemic risk factors [82,83].

### 3.8. Oral Candidiasis in Malnutrition

Oral candidiasis is an opportunistic infection rather than a primary deficiency lesion. The bibliography indicates that malnutrition can increase susceptibility and worsen the clinical course. This relationship appears especially in older, hospitalized, medically fragile, or immunologically compromised populations, in whom nutritional depletion, salivary dysfunction, denture wearing, and antimicrobial exposure frequently coexist [84,85]. Recent pediatric research found lower levels of iron, zinc, albumin, vitamin D, and vitamin A in children with oral candidiasis than in controls [86,87,88,89]. Although these outcomes do not prove causality, they fit a reliable biological model in which nutritional compromise weakens mucosal and salivary defenses, resulting in fungal overgrowth [90].

Clinically, candidiasis may coexist with angular cheilitis, glossitis, denture stomatitis, mucosal erythema, burning sensation, and taste disturbance [91]. In case of recurrent or refractory candidiasis without an obvious precipitating factor, the literature supports considering malnutrition, micronutrient deficiency, and systemic disease in the differential diagnosis [92].

Although much of the research focuses on discrete micronutrient deficiencies, several sources broaden the discussion to protein-energy malnutrition, hospitalized patients, and eating disorders such as anorexia or bulimia nervosa [93,94,95,96]. These conditions matter because they can create combined deficits that affect saliva, immunity, mucosal turnover, and food tolerance [97,98].

Protein-energy malnutrition in childhood has been linked to diminished salivary flow and altered oral function later in life, suggesting a long-term effect on exocrine defense systems [12,33,99]. Reviews on anorexia nervosa, bulimia nervosa, and eating disorders also describe oral soft tissue trauma, ulceration, xerostomia and candidal susceptibility [100,101].

These broader malnutrition states are especially relevant when patients present with multiple concurrent signs rather than a single classic lesion. Oral soreness, reduced salivary protection, traumatic ulceration, erythema, commissural lesions, and fungal infection may together reflect inadequate intake, malabsorption, self-induced vomiting, or chronic systemic undernutrition [102,103,104].

### 3.9. Differential Diagnosis and Clinical Interpretation

One of the main difficulties in diagnosing malnutrition-related oral mucosal disease is the low specificity of individual lesions. Recurrent ulcers may reflect such conditions as aphthous disease, Behcet disease, celiac disease, inflammatory bowel disease, drug reactions, autoimmune conditions, hematologic disorders, or local trauma, while glossitis and oral burning may be caused by candidiasis, xerostomia, neuropathy, endocrine dysfunction, contact allergy, or idiopathic pain syndromes [9,105,106,107,108]. For that reason, the clinical value of oral manifestations lies less in their specificity than in their pattern recognition. The combination of recurrent aphthae, atrophic tongue, angular cheilitis, mucosal pallor, burning, dysgeusia, or candidiasis should heighten suspicion of iron, folate, vitamin B12, or zinc deficiency, particularly when accompanied by fatigue, gastrointestinal symptoms, autoimmune history, restricted diet, metformin exposure, gastric surgery, or advanced age [10,109].

The literature also emphasizes that deficiency-related oral findings may precede anemia or macrocytosis [53,110]. Therefore, normal hemoglobin alone does not exclude a nutritional contribution, and more targeted testing may still be warranted when the phenotype is suggestive [53,110,111].

### 3.10. Nutrition in Periodontal Disease

Although the primary focus of this review is on malnutrition and oral mucosal diseases, it is important to acknowledge the broader relationship between nutrition and periodontal health. Growing evidence indicates that dietary factors and micronutrient status influence the development and progression of periodontal disease. Vitamins C, D, and E, essential fatty acids, coenzyme Q10, and probiotics have been shown to modulate inflammatory responses, support connective tissue integrity, and potentially improve clinical periodontal outcomes [99].

At the same time, key periodontal pathogens have evolved sophisticated mechanisms to exploit host nutrients, particularly iron and heme. *Porphyromonas gingivalis*, a keystone pathogen in chronic periodontitis, is a heme auxotroph that cannot synthesize heme de novo and therefore relies entirely on host-derived heme as a source of iron and protoporphyrin IX [112,113,114]. To acquire these nutrients, *P. gingivalis* employs multiple virulence factors, including cysteine proteases (gingipains), hemagglutinins, hemolysins, and dedicated heme acquisition systems such as Hmu and Hus. Gingipains degrade host hemoglobin and other heme-containing proteins, releasing heme that is subsequently bound by surface hemophores (e.g., HmuY) and transported into the bacterial cell via the Hmu receptor complex. In addition, *P. gingivalis* can utilize inorganic iron through the FeoB transporter and may activate manganese transport as a compensatory mechanism under iron-limited conditions [112].

These microbial strategies highlight a bidirectional relationship: while host nutritional status influences periodontal inflammation and tissue resilience, periodontal pathogens actively compete for essential nutrients, potentially altering local and systemic nutrient homeostasis. However, a detailed discussion of periodontal disease and microbial nutrient acquisition is beyond the scope of the present review, which is focused on malnutrition-related oral mucosal manifestations.

## 4. Diagnostic Work-Up

The previous studies strongly support laboratory screening in patients with persistent, recurrent, multifocal, or unexplained oral mucosal symptoms compatible with nutritional deficiency. A full diagnostic process shall include a complete blood count, hemoglobin indices, serum ferritin or iron levels, vitamin B12, folate, and, when clinically appropriate, zinc and homocysteine [53,110,115,116]. In selected cases, evaluation for gastric parietal cell antibodies, malabsorption, celiac disease, inflammatory bowel disease, or medication-associated deficiency may be justified [15,110]. This diagnostic strategy is particularly relevant for recurrent aphthous stomatitis, atrophic glossitis, burning mouth symptoms, and angular cheilitis because these are the conditions most frequently associated with hematinic abnormalities [11,44,61]. A detailed dietary and medical history is equally important, since low intake, alcoholism, restrictive diets, gastric surgery, pernicious anemia, metformin use, and chronic gastrointestinal disease may all contribute to oral presentations of deficiency [15,117].

Given the frequent association between oral manifestations and systemic disease, a multidisciplinary approach is often required. Depending on the clinical and laboratory findings, collaboration with other specialists, particularly gastroenterologists, hematologists, and clinical nutritionists, may be essential to identify malabsorption disorders, systemic diseases, or dietary causes and to ensure appropriate management. In patients with unexplained or recurrent oral mucosal symptoms, referral for gastrointestinal evaluation (e.g., celiac serology, endoscopy) and detailed nutritional assessment should be considered as part of a comprehensive diagnostic strategy [9,10,15,109,118].

## 5. Management Implications

Management of malnutrition-related oral mucosal disease requires systemic treatment, not only symptomatic relief. Topical corticosteroids, antiseptics, local anesthetics, or saliva substitutes may reduce discomfort, but sustained improvement often depends on identifying and correcting the underlying problem [116,118,119].

For recurrent aphthous stomatitis, classical and contemporary studies suggest that supplementing with vitamin B12, folate, or iron can reduce recurrence or improve symptoms in selected patients [21,120]. Zinc supplementation has been effective for the treatment of recurrent aphthae, burning mouth syndrome, and oral lichen planus, with some favorable clinical reports [28,37,64,65,75]. Atrophic glossitis and deficiency-associated burning symptoms may improve substantially once iron, folate, or vitamin B12 depletion is corrected, particularly when the causative factor is recognized early [59,65]. Some articles discussed probiotic supplementation for the treatment of oral candidiasis, but results remain inconclusive and require further research [121].

Management should also address the cause of the deficiency rather than the laboratory value alone. Patients with pernicious anemia, gastric autoimmunity, celiac disease, post-bariatric malabsorption, eating disorders, or medically complex frailty require interdisciplinary care. Oral lesions may recur if the systemic disorder remains untreated [74].

Practical information on the reference ranges, routes of administration, commonly used dosage regimens, treatment duration, monitoring parameters, and relevant precautions for vitamin B12, folate, zinc, and iron replacement is summarized in Table 1.

## 6. Limitations of the Evidence

The literature available has several limitations that should temper interpretation. Much of the evidence consists of cross-sectional, case–control, or retrospective observational studies, which are useful for identifying associations but cannot consistently determine causality. Patient populations, diagnostic criteria, laboratory thresholds, and control selection also vary across studies, making direct comparisons difficult [5,17,44].

Another limitation is that oral mucosal diseases are multifactorial. Nutritional deficiency may be causal in some patients, contributory in others, and incidental in still others, especially in disorders such as oral lichen planus or burning mouth syndrome where immune, neuropathic, psychological, and microbial pathways also matter. Nevertheless, the repeated identification of hematinic deficits across multiple oral phenotypes supports their clinical relevance even when they are not the sole pathogenic driver [5,10,44,54].

Finally, this review is intentionally narrative. The goal of this article is to clinically synthesize the literature around an oral medicine question, but a formal systematic review with explicit database searching and risk-of-bias assessment would be required to quantify the evidence more rigorously.

## 7. Conclusions

The literature clearly states that nutritional deficiencies lead to different types of lesions of the oral mucosa. As proven in the article, the most common mucosal expressions of malnutrition involve recurrent ulcerations, atrophic glossitis, burning sensations, angular cheilitis, mucosal erythema, lingual surface change and opportunistic candidal infection as presented in Table 2. Iron, vitamin B12, folate, and zinc are the most consistently implicated deficiencies, while vitamins A, C, and D may have adjunctive or situation-dependent roles through effects on epithelial repair, oxidative stress, and immune regulation.

The strongest clinical association with nutritional deficiency is seen among the reviewed conditions in recurrent aphthous stomatitis and atrophic glossitis, followed by burning mouth presentations and some cases of angular cheilitis or candidiasis. The main practical implication is clear: persistent or unexplained oral mucosal disease should encourage consideration of nutritional screening, because the mouth may reveal systemic deficiency before broader signs become obvious. In this way, the oral examination is not only diagnostic of local disease but also an opportunity to detect reversible systemic malnutrition early.

## Figures and Tables

**Table 1:** Reference ranges for the main laboratory parameters and treatment algorithm.

	Reference Ranges	Route of Administration	Recommended Dosage	Treatment Duration	Monitoring Parameters	Relevant Precautions
Vit. B_12_	Serum concentration 150–500 pmol/L (200–575 ng/L) [122] In anemia, MCV may be elevated >100 fl.	Intramuscular or deep subcutaneous injection or oral (tablets, effervescent tablets, drops, syrups, oral sprays, capsules, etc.) [122]. I.M.—in severe cases—e.g., psychiatric, neurologic or in pregnant women	Intramuscular/ subcutaneous administration—1000–2000 µg 1xday Oral administration 1000–2000 µg 1xday [122].	I.m./S.c.— 7–14 days 1xday, and then 1x/week for 4–8 weeks. Maintenance phase: s.c. 1000 µg 1×/month or 1×/3 months or 1000–2000 µg/d p.o	Serum vitamin B_12_ concentration > 150 pmol/L (200 ng/L) [122] After 7 days, Hb level should be elevated.	Population groups at high risk of Vit. B_12_ deficiency: vegetarians, vegans, the elderly, pregnant, and lactating women [122].
Folate	[in serum] 6,8–26 nmol/L (3–12 μg/L) (on empty stomach) [in erythrocytes] 520–1600 nmol/L (235–721 μg/L) Also homocysteine concentration determines folate deficiency if: [in serum] homocysteine <4 ng/mL (<10 nmol/L) [in erythrocytes] <151 ng/mL (<340 nmol/L) [123].	Oral supplementation [124]	1–5 mg/day [124]	1–4 months (until normalisation of blood indices) or as long as the reason of deficiency is present [124].	Correct blood results, serum folate result over 6.8 nmol/L [123].	Serum folate shows important variation, reflecting recent food intake, while red blood cell folate remains stable, showing average body folate status at the time of the production of the population of red cells. If accompanied with vit. B_12_ deficiency, needs to be treated simultaneously, to avoid neurological exacerbation [124].
Zinc	Serum levels: Adults: 60–120 µg/dL (9–18 µmol/L), children: 11–24 µmol/L (70–160 µg/dL) Diagnosis of zinc deficiency is mostly based on clinical symptoms. Laboratory zinc values are less reliable in the diagnosis of zinc deficiency due to zinc sequestration in tissues caused by stress, infection, illness, or decreased serum concentration.	Change of dietary habits + Oral supplementation [125].	Usually 25 mg; maximum daily dose: 40 mg [125]		Mainly elimination of symptoms of deficiency	High zinc intake levels can result in adverse effects, including nausea, vomiting, loss of appetite, abdominal cramps, diarrhea and interference with the body’s absorption of essential minerals, such as iron and copper [125].
Iron	Iron deficiency is diagnosed by low serum ferritin (typically <30 ng/mL) in individuals without inflammatory conditions + TSAT (transferrin saturation) <20% [126]	Oral administration: Iron salts (such as ferrous sulfate, ferrous gluconate, ferrous fumarate, and ferric citrate) on empty stomach (at least 1 h before meal) Intravenous administration: in most severe cases [127], e.g., oral iron intolerance, poor absorption, chronic inflammatory conditions [126].	Oral: Traditional guidance: 150–200 mg split into 2–3 doses Recent guidance: daily oral dose ≤ 40 mg or ≥60 mg on alternate days (High doses of oral iron increase hepcidin levels, which inhibit iron absorption for up to 24 h. Intake at 48 h intervals improves iron absorption). I.V.—up to 1000 mg in short (15–20 min.) infusion [127].	Until Hb levels normalize, and then continuation for about 3 months to restore iron stores completely (even up to 6 months) [127].	Monitoring Hb levels during first 4 weeks to evaluate the response. Treatment is considered successful when serum ferritin levels reach 100 µg/L [127].	Serum ferritin level is affected by inflammatory conditions—may be falsely elevated. Unabsorbed iron can cause intestinal irritation, inflammation, and dysbiosis [127].
Hb	Male: 13–16.5 g/dL (8.7–10.2 mmol/L) Female: 12–16 g/dL (7.5–9.9 mmol/L), pregnant: 11–14 g/dL (6.9–8.8 mmol/L)	-	-	-	-	-

**Table 2:** Nutrition deficiency linked to oral mucosa condition.

Oral Mucosa Condition	Linked Nutrition Deficiency	Description
Recurrent Aphthous Stomatitis	Iron, folate, vitamin B12 [2,5,41], vitamin D [44,45,47]	Recurrent painful ulceration, often linked to hematinic deficits.
Atrophic Glossitis	Iron, folate, vitamin B12, protein-energy malnutrition [21,22,53,54,57,59]	Smooth, red, sore tongue caused by mucosal atrophy.
Burning Mouth Symptoms/Glossodynia	Vitamin B12, B2, B6, zinc, iron, folate [58,63,64]	Burning or painful oral sensation, mostly with no visible change
Angular Cheilitis	Iron, folate, B-vitamin; protein-energy malnutrition [66,68]	Fissuring and inflammation at the mouth corners.
Stomatitis	B-vitamin, iron, protein-energy malnutrition [12,13,31]	General inflammation of the oral mucosa.
Mucosal Erythema	Vitamin B12, folate, iron [10,93,128]	Redness of oral mucosa often seen with epithelial atrophy or inflammation.
Oral Lichen Planus and Related Inflammatory Mucosal Disease	Zinc, vitamin B12, hemoglobin, iron, folate, high homocysteine level [62,73,75,76,77,78]	Nutritional factors may worsen inflammatory mucosal disease.
Geographic Tongue and Lingual Morphologic Change	B-vitamin, iron deficiency, zinc deficiency [79,80,81,82]	Altered tongue surface pattern or papillary changes.
Oral Candidiasis	Vitamin D, iron, zinc, albumin, vitamin A [84,85,86,129,130]	Opportunistic fungal overgrowth favored by impaired host defense.

## Data Availability

No new data were created or analyzed in this study. Data sharing is not applicable to this article.
